# Complementary biomarkers of computed tomography for diagnostic grading of gastric cancer: DSCC1 and GINS1

**DOI:** 10.18632/aging.205491

**Published:** 2024-01-31

**Authors:** Yufeng Zhu, Shiyang Hou, Chunbo Kang

**Affiliations:** 1 Department of Radiology, The First People’s Hospital of Fuyang, Fuyang, Hangzhou 311400, China; 2 Department of General Surgery, Beijing Rehabilitation Hospital Affiliated to Capital Medical University, Shijingshan, Beijing 100144, China

**Keywords:** gastric cancer, DSCC1, GINS1, biomarkers, computed tomography

## Abstract

Objective: Computed tomography (CT) is an important tool for grading gastric cancer. Gastric cancer typically originates from epithelial cells of gastric mucosa. However, complementary markers for gastric cancer, relationship between DSCC1, GINS1 and gastric cancer remain unclear.

Methods: Gastric cancer data were obtained from gene expression omnibus (GEO). Differentially expressed genes (DEGs) were identified, weighted gene co-expression network analysis (WGCNA) was conducted. Protein-protein interaction (PPI) network was constructed and analyzed. Functional enrichment analysis, gene set enrichment analysis (GSEA), gene expression heatmaps, immune infiltration analysis were performed. The most relevant diseases related to core genes were identified using Comparative Toxicogenomics Database (CTD). TargetScan was used to screen miRNAs. Validation was carried out using Western blotting (WB) and reverse transcription-polymerase chain reaction (RT-PCR).

Results: 1243 DEGs were identified. Gene ontology (GO) and Kyoto Encyclopedia of Gene and Genome (KEGG) analyses revealed significant enrichment in cell cycle regulation, macrophage migration control, basement membrane, extracellular regions, growth factor binding, protein complex binding, P53 signaling pathway, protein digestion and absorption, metabolic pathways. Immune infiltration analysis indicated that high expression of activated Mast cells and Neutrophils, with a strong positive correlation between them, may influence progression of gastric cancer. CTD analysis revealed associations between DSCC1, GINS1 and gastric tumors, gastrointestinal diseases, tumors, gastritis, inflammation, necrosis. WB and RT-PCR results demonstrated high expression of DSCC1 and GINS1 in gastric cancer.

Conclusion: The expressions of DSCC1 and GINS1 are up-regulated in gastric cancer, which can be used as supplementary markers for CT diagnostic grading of gastric cancer.

## INTRODUCTION

Computed tomography (CT) plays a crucial role in the diagnosis, staging, and follow-up of gastric cancer. Gastric cancer, a malignant tumor typically originating from cells within the gastric mucosa layer, initially forms as small tumors and may subsequently spread to surrounding tissues and organs [1]. It can be classified into different subtypes, including adenocarcinoma, infiltrative adenocarcinoma, mucinous adenocarcinoma, among others. Gastric cancer is a global cancer with varying incidence rates across different countries and regions. Epidemiological studies indicate higher incidence rates in Asia, Latin America, and Eastern Europe, while lower rates are observed in North America and Western Europe. Gastric cancer is characterized by its heterogeneity, with different subtypes exhibiting distinct features [2]. CT is valuable in staging gastric cancer, which involves determining the extent of the disease and whether it has spread to nearby organs or lymph nodes. CT scans can help evaluate the size and location of the tumor and assess its involvement with adjacent structures.

Clinical presentations of Gastric cancer may vary among individuals, but common symptoms include indigestion, upper abdominal discomfort, loss of appetite, weight loss, vomiting, and black stools. These symptoms often appear in advanced stages, underscoring the importance of early diagnosis for successful treatment [3]. Pathological characteristics of Gastric cancer include tumor type, grading, depth of invasion, lymph node metastasis, and the morphological features of tumor cells. These characteristics are vital for assessing the severity of the condition and determining appropriate treatment strategies. Without timely diagnosis and treatment, Gastric cancer can rapidly spread to surrounding tissues and organs, posing a significant threat to the patient’s life [4, 5]. It can impact a patient’s appetite and nutritional intake, leading to weight loss and weakness.

To date, the exact causes of Gastric cancer remain unclear. However, certain risk factors have been identified, including dietary habits, Helicobacter pylori infection, smoking, family history, and genetic factors. Understanding the molecular mechanisms of Gastric cancer is crucial for gaining insights into its pathogenesis and developing more effective treatment strategies. CT scans are essential for detecting distant metastases, especially to organs such as the liver, lungs, and peritoneum. This information is crucial for determining the overall stage of the disease and planning appropriate treatment.

In recent years, the field of bioinformatics has played a pivotal role in advancing research. It enables in-depth investigations at the genomic, proteomic, and metabolic levels, helping reveal new biological knowledge and potential drug targets [6]. These advances contribute to the realization of precision medicine, making healthcare more personalized and capable of predicting disease risks and tailoring treatment plans to individual patients [7].

DSCC1 and GINS1 are two genes or proteins associated with DNA replication and cell cycle regulation [8, 9]. DSCC1 is involved in the process of DNA replication and sister chromatid cohesion. It acts as an accessory protein, working in collaboration with other replication and cohesion proteins to ensure the smooth replication and segregation of DNA during cell division. GINS1 is a component of the GINS complex, which plays a significant role in DNA replication and cell division, similar to DSCC1. In recent years, bioinformatics techniques have been employed in Gastric cancer research to identify potential therapeutic targets, such as HER2, PD-L1, and others [10], all of which have demonstrated a certain degree of regulatory impact on gastric tumors. However, the exact relationship between DSCC1, GINS1, and Gastric cancer is not yet clear. However, complementary markers of computed tomography for diagnostic grading of gastric cancer are currently unclear, and the relationship between DSCC1 and GINS1 and gastric cancer remains unclear.

Therefore, this study aims to use bioinformatics techniques to screen complementary markers of computed tomography for diagnostic grading of gastric cancer, conducting enrichment analysis and pathway analysis. DSCC1 and GINS1’s significant roles in gastric cancer will be validated using public datasets. Furthermore, basic cellular experiments will be applied to validate their functions.

## METHODS

### Gastric cancer dataset

In this study, Gastric cancer datasets GSE79973, GSE118916, GSE118897, and GSE172032 were downloaded from the Gene Expression Omnibus (GEO) database (http://www.ncbi.nlm.nih.gov/geo/). GSE79973 includes 10 Gastric cancer and 10 normal tissue samples, GSE118916 includes 15 Gastric cancer and 15 normal tissue samples, GSE118897 includes 10 Gastric cancer and 10 normal tissue samples, and GSE172032 includes 4 Gastric cancer and 4 normal tissue samples. These datasets were used to identify differentially expressed genes (DEGs).

### Batch removal

For data integration and batch removal, the R package “inSilicoMerging” was first used to merge the datasets GSE79973, GSE118916, GSE118897, and GSE172032, generating a merged matrix. Further batch effect removal was performed using the “removeBatchEffect” function from the R package “limma” (version 3.42.2).

### DEG selection

The R package “limma” was employed for probe summarization and background correction of the merged matrix from GSE79973, GSE118916, GSE118897, and GSE172032. The Benjamini-Hochberg method was used to adjust the raw *p*-values, and the fold change (FC) was calculated using the false discovery rate (FDR). DEGs were selected based on the criteria of *p* < 0.05 and FC >1.5, and a volcano plot was generated to visualize DEGs.

### Weighted gene co-expression network analysis (WGCNA)

WGCNA was performed using the merged and batch-corrected gene expression matrix from GSE79973, GSE118916, GSE118897, and GSE172032. First, the median absolute deviation (MAD) of each gene was calculated, and the lowest 50% of genes with the smallest MAD were removed. The “goodSamplesGenes” function from the R package WGCNA was used to remove outlier genes and samples. The scale-free co-expression network was constructed using WGCNA. A power function A_mn = |C_mn|^β was employed to create a weighted adjacency matrix, where C_mn represents the Pearson correlation between Gene_m and Gene_n, and A_mn represents the adjacency between Gene_m and Gene_n. β is a soft thresholding parameter that emphasizes strong correlations between genes and diminishes the impact of weak and negative correlations. A power of 10 was chosen, and the adjacency matrix was transformed into a topological overlap matrix (TOM) to measure the connectivity of genes. Modules were defined using hierarchical clustering based on the TOM-based dissimilarity. The minimum module size was set to 30 genes, and a sensitivity of 3 was applied. Modules with a dissimilarity of less than 0.25 were merged, with the “grey” module representing unassigned genes.

### Functional enrichment analysis

Gene Ontology (GO) and Kyoto Encyclopedia of Genes and Genomes (KEGG) analyses were conducted to assess gene function and biological pathways. The enrichment analysis was performed using the R package “clusterProfiler” based on gene lists generated from the Venn diagram. The most recent KEGG pathway gene annotations were obtained by inputting the DEG list to the KEGG REST API (https://www.kegg.jp/kegg/rest/keggapi.html). The enrichment analysis was conducted with a background of the gene list and set significance criteria with a minimum gene set size of 5, a maximum gene set size of 5000, a *p*-value of < 0.05, and an FDR of < 0.25. Additionally, the Metascape database was used for comprehensive gene list annotation and analysis.

### Gene set enrichment analysis (GSEA)

For Gene Set Enrichment Analysis (GSEA), samples were divided into two groups, disease and normal, based on the phenotype. The gene set “c2.cp.kegg.v7.4.symbols.gmt” was downloaded from the Molecular Signatures Database to assess relevant pathways and molecular mechanisms. GSEA software (version 3.0) was used to perform GSEA based on gene expression profiles, with parameters set to a minimum gene set size of 5, a maximum gene set size of 5000, 1000 permutations, a *p*-value of < 0.05, and an FDR of < 0.25. GO and KEGG analyses were also performed on the entire gene list.

### Immune infiltration analysis

CIBERSORT, a widely used method for estimating immune cell infiltration, was applied to the merged and batch-corrected gene expression matrix of the Gastric cancer datasets GSE79973, GSE118916, GSE118897, and GSE172032. The CIBERSORT algorithm was used to deconvolve the immune cell subtype expression matrix, and samples with a confidence *p*-value of < 0.05 were selected.

### Protein-protein interaction (PPI) network construction and analysis

The Search Tool for the Retrieval of Interacting Genes (STRING) database (http://string-db.org/) was used to construct a PPI network of the identified core genes. Cytoscape software was employed for network visualization and to predict core genes. MCODE was used to find the best modules within the PPI network. Additionally, the top ten genes with the highest correlation using four different algorithms (MCC, DMNC, EPC, Radiality) were identified and the intersection was considered as the core gene list.

### Survival analysis

Clinical survival data for Gastric cancer were obtained from TCGA. The “maxstat” function in R was used to calculate the optimal cutoff value for the RiskScore of core genes. Patients were divided into high and low-risk groups based on the cutoff value, and survival differences were analyzed using the “survfit” function in R with the log-rank test to evaluate the significance of survival differences between sample groups.

### Gene expression heatmaps

Gene expression heatmaps were generated using the R package “heatmap” for the core genes identified in the PPI network, showing the expression differences in GSE79973, GSE118916, GSE118897, and GSE172032 after batch correction and merging.

### CTD analysis

The Comparative Toxicogenomics Database (CTD) was used to identify the most relevant diseases associated with the core genes. Radar plots of gene expression differences for each gene were created using Excel.

### miRNA analysis

TargetScan (https://www.targetscan.org) was utilized to predict miRNAs that may regulate the identified DEGs. In this study, TargetScan was used to screen miRNAs that potentially regulate the central DEGs.

### The experimental assays

#### Cell lines and Western blotting (WB)

The normal gastric mucosal epithelial cells (NGEC, GES-1, CAT#STCC10401G, Servicebio, Wuhan) and gastric cancer cells (HGC-27, CAT#STCC10403G, Servicebio, Wuhan) were cultured. We set up control group (normal gastric mucosal epithelial cells), gastric cancer cell group, gastric cancer DSCC1 gene overexpression group (Gastric cancer-DSCC1_OE) and gastric cancer DSCC1 gene knockout group (Gastric cancer-DSCC1_KO), and carried out Western blotting experiments in four groups.

To construct DSCC1 downregulated cell line, small interference (si) RNAs against DSCC1 (DSCC1-Hu- 396 CCAAGUUAAAGAAGCUAAAGAUUUAGCUUCUUUAACUUGGGU) was transfected into HGC-27 cells. The pcDNA3.1-DSCC1 (>NM_024094.3: 100-1281) vector was transfected into HGC-27 cells to construct DSCC1 overexpression cell line. Transfection process was conducted using SweTransRNA transfection reagent (CAT# G1806, Servicebio, Wuhan) as per manufacturers’ directions. After transfection for 24 h, cells were collected to conduct follow-up experiments. All the si-RNAs and plasmids were synthesized by Shanghai GenePharma Co., Ltd (Shanghai, China).

Sample preparation (total protein): Place the cell culture dish on ice and wash the cells three times with ice-cold PBS. Aspirate the PBS, then add ice-cold RIPA buffer (Protease and phosphatase inhibitors should be added before use). Scrape adherent cells off the dish, then gently transfer the cell suspension into a 1.5mL microcentrifuge tube. Maintain constant agitation for 30 min in ice bath, use pipette to mix it up. Centrifuge at 12,000 rpm, 4°C for 10 min. Gently remove the tubes from the centrifuge and place on ice, tranfer the supernatant to a fresh tube. Protein quantification was performed using the BCA protein quantification reagent kit, and a standard curve was generated using a spectrophotometer. Protein separation: The quantified proteins were separated using SDS-Polyacrylamide Gel Electrophoresis (SDS-PAGE). Transfer to PVDF membrane: The proteins were then transferred to a Polyvinylidene Fluoride (PVDF) membrane. Blocking: The membrane was blocked using 5% Bovine Serum Albumin (BSA) at room temperature for 1 hour. Antibody incubation: Subsequently, the membrane was incubated with specific antibodies, including DSCC1 antibody, GINS1 antibody, and GAPDH antibody, to detect the respective proteins. GAPDH was used as an internal reference. Washing and secondary antibody incubation: The membrane was incubated with the respective secondary antibodies (diluted at 1:5000) at room temperature for 60 minutes after washing the membrane with TBST three times, each for 5-10 minutes. Chemiluminescence detection: After washing off excess solution from the PVDF membrane and placing it on absorbent paper, the membrane was submerged in a mixture of Enhanced Chemiluminescence (ECL) reagent. After a 1-minute incubation, the membrane was placed in a chemiluminescence imager, and chemiluminescence was initiated according to the preset program. The original data were saved, and subsequent data analysis was performed using AIWBwellTM analysis software.

### Reverse transcription-polymerase chain reaction (RT-PCR)

RNA-Quick Purification Kit (ESscience Biotech, CAT#RN001), Fast All-in-One RT Kit (with gDNA Remover) (ESscience Biotech, CAT#RT001) and 2×Taq PCR MasterMix (Biomed, CAT#MT201) kits were used.

The RT-PCR grouping is consistent with the Western blot (WB) experiments. The specific operational steps are as follows: a suspension containing 1×10^6 cells is transferred to a 1.5 ml centrifuge tube and centrifuge at 500×g in an Eppendorf centrifuge (Model 5810R, Germany) for 3 min to precipitate the cells. Then add 500 μl of lysis buffer, pipette vigorously 10 times and vortex for 10 seconds. Add equal volumes of anhydrous ethanol to the lysed cells and mix the mixture thoroughly. Invert the tube several times or pipette vigorously 10 times to disperse any potential precipitate. The liquid is then transferred to a centrifugal column and centrifuged for 1 minute. Next, remove the genomic DNA. Take total RNA 100 ng ~ 2 μg and add DNase 2 μl. Add water to a final volume of 16 μl and gently pipette 5-10 times to mix. The mixture is reacted at room temperature (about 25°C) for 5 minutes and then placed on ice. The concentration and purity of RNA are detected by ultramicro- spectrophotometer (NanoDrop2000, Thermo). Take 16 μl volume of DNAzyme-treated total RNA, add 4 μl 5×RT Mix (ES Science, RT001, Shanghai, China), Pipette 10 times to mix thoroughly, incubate for 15 min at 42°C and dilute 5-10 times as PCR template. PCR amplification proceeds through a series of temperature cycles, involving denaturation, annealing, and extension steps. Primer sequences are as follows: DSCC1 Forward 5’TCGTGGTGATAAAGACGAGCA3’, Reverse 5’CCGGAGTTTTACAACCAGGAAT3’; GINS1 Forward 5’ACGAGGATGGACTCAGACAAG3’, Reverse 5’TGCAGCGTCGATTTCTTAACA3’; GAPDH Forward 5’GGAGCGAGATCCCTCCAAAAT3’, Reverse 5’GGCTGTTGTCATACTTCTCATGG3’. After the amplification is completed, a final extension step is often carried out to ensure that all DNA strands have been fully synthesized.

### Data availability

The datasets generated during and/or analyzed during the current study are available from the corresponding author on reasonable request.

## RESULTS

### Differential gene expression analysis

**Figure 1:**
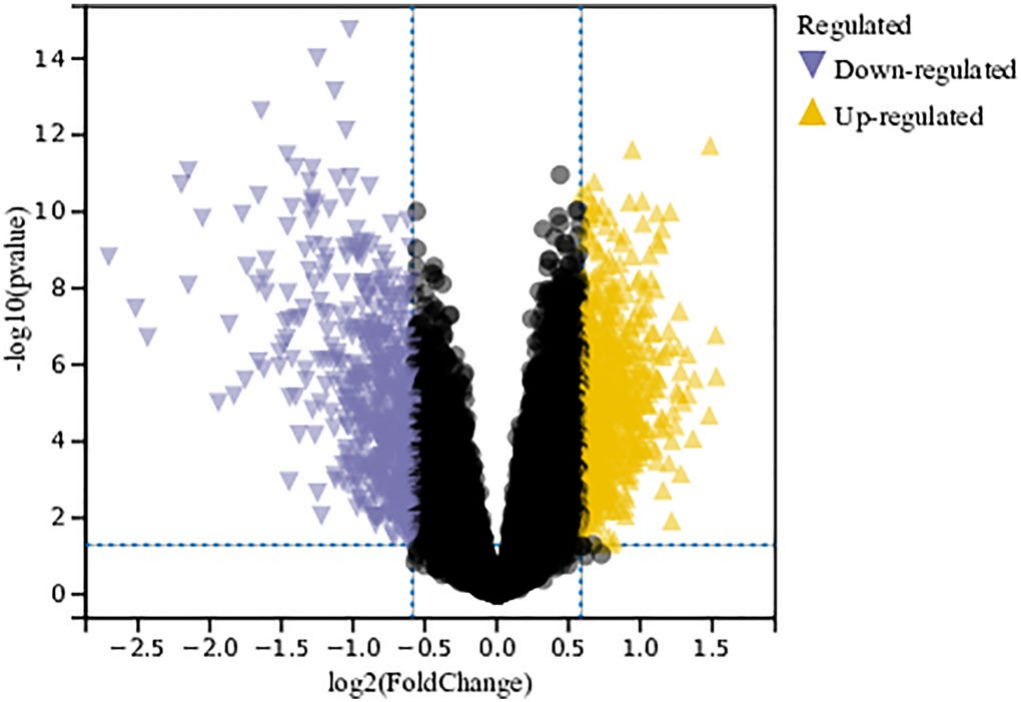
**Analysis of differentially expressed genes.** A total of 1243 DEGs were identified.

In this study, differential gene expression was identified in the merged and batch-corrected matrices of Gastric cancer datasets GSE79973, GSE118916, GSE118897, and GSE172032 using a predefined cutoff value. In total, 1243 DEGs were identified (Figure 1).

### Functional enrichment analysis

#### DEGs

**Figure 2:**
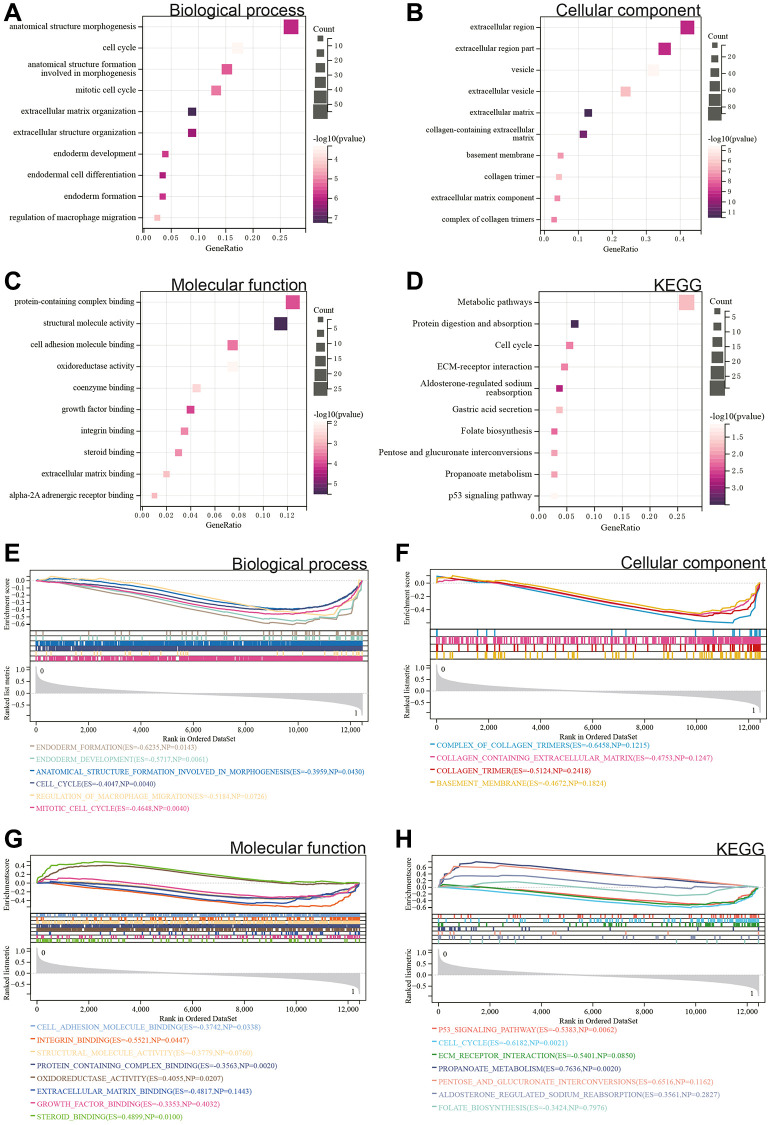
**Functional enrichment analysis.** (**A**–**D**) DEGs. (**E**–**H**) GSEA.

We performed GO and KEGG analyses on these DEGs. According to the GO analysis, in the Biological Process (BP) category, DEGs were mainly enriched in the cell cycle and regulation of macrophage migration (Figure 2A). In the Cellular Component (CC) category, DEGs were mainly enriched in the basement membrane and extracellular region (Figure 2B). In the Molecular Function (MF) category, they were primarily involved in growth factor binding and protein complex binding (Figure 2C). In the KEGG analysis, DEGs were enriched in the P53 signaling pathway, protein digestion and absorption, and metabolic pathways (Figure 2D).

#### GSEA

Additionally, a GSEA was conducted on the entire genome to identify potential enrichments among non-differentially expressed genes and validate the results obtained from DEGs. The intersection of enrichment terms with GO and KEGG enrichments for the merged Gastric cancer dataset indicated that DEGs were significantly enriched in the cell cycle, regulation of macrophage migration, protein complex binding, and the P53 signaling pathway (Figure 2E–2H).

#### Metascape enrichment analysis

**Figure 3:**
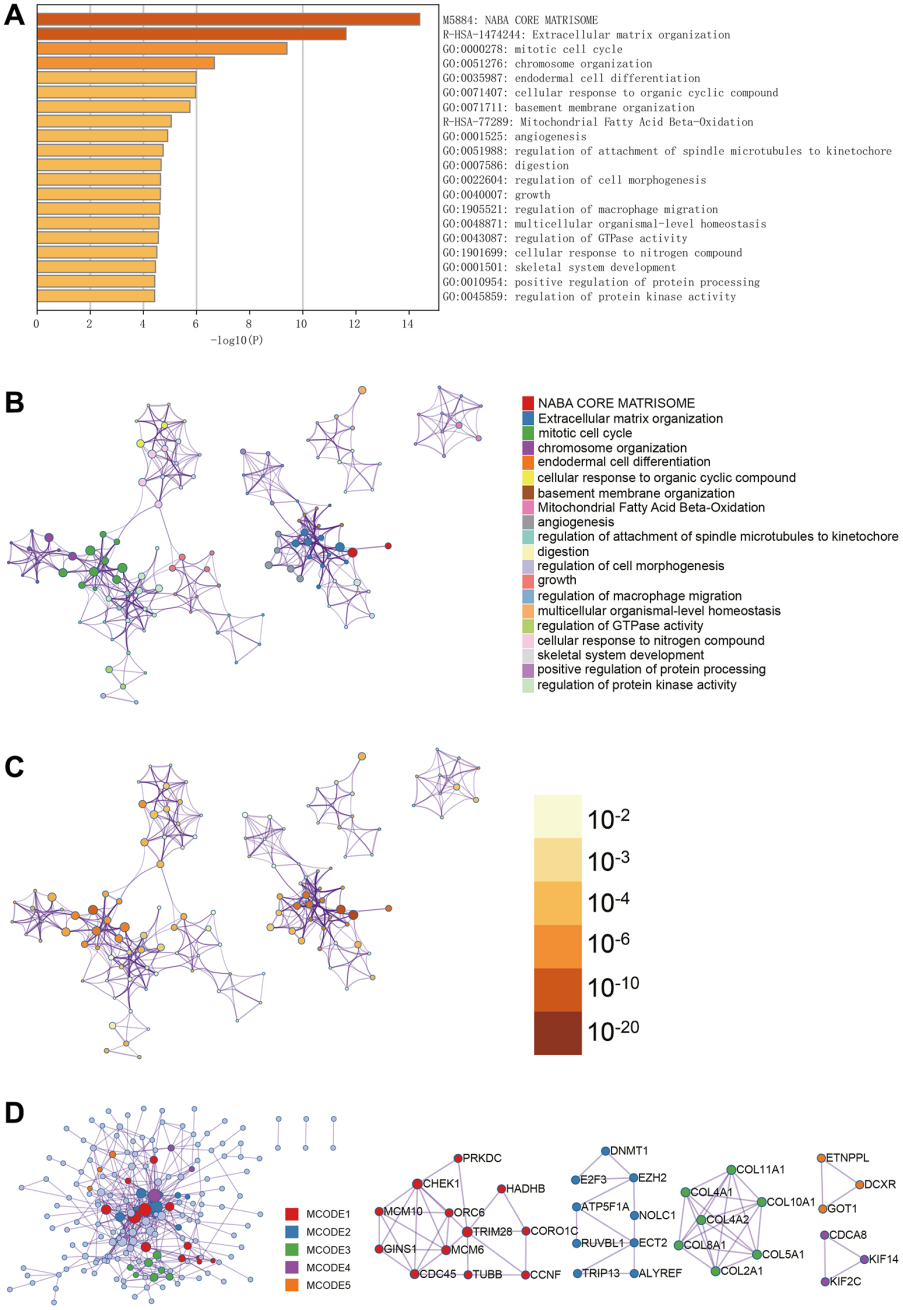
**Metascape enrichment analysis.** (**A**) GO enrichment highlights macrophage migration control and mitotic cell cycle. (**B**–**D**) Output the enrichment network colored by enrichment terms; output the enrichment network colored by *p*-value; visualize the association and confidence representing each enrichment item.

In Metascape’s enrichment analysis, the GO enrichment terms included the regulation of macrophage migration and mitotic cell cycle (Figure 3A). Enrichment networks colored by enrichment terms and *p*-values were visualized (Figure 3B–3D), providing a representation of associations and confidence levels for each enrichment term.

#### Immune infiltration analysis

**Figure 4:**
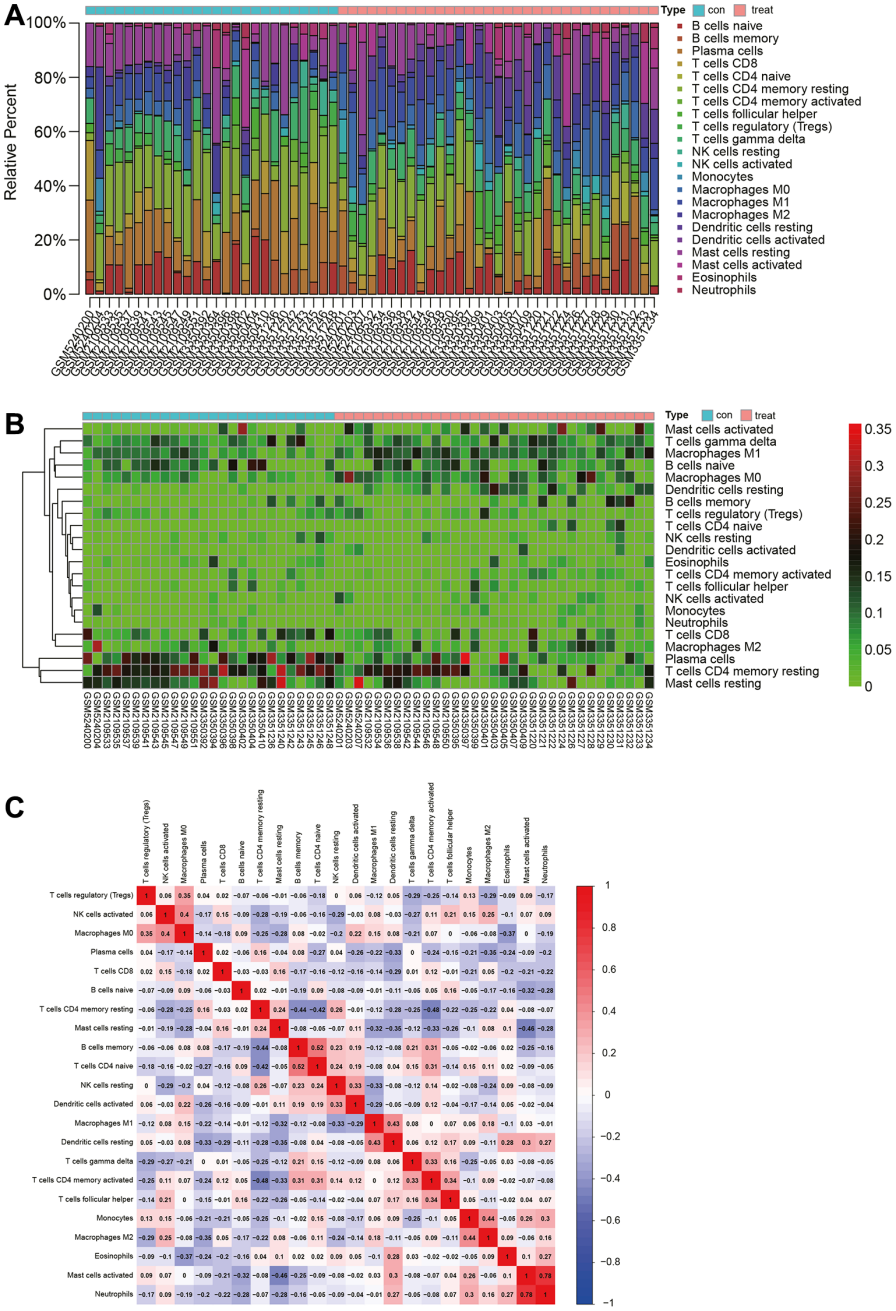
**Immune infiltration analysis.** (**A**) Proportion of naive CD4 T cells in samples. (**B**) Heatmap of immune cell expression levels. (**C**) Analysis of immune cell correlations.

We used the CIBERSORT package to analyze the merged matrix of Gastric cancer datasets. At a 95% confidence level, the proportions of immune cells in the whole gene expression matrix were estimated. The results indicated a relatively high proportion of T cells CD4 naive in the samples (Figure 4A). An immune cell expression heatmap for the dataset was also generated (Figure 4B). Furthermore, correlation analysis was conducted on infiltrating immune cells, revealing that when Mast cells activated were highly expressed, Neutrophils also showed elevated expression. A strong positive correlation between Neutrophils and Mast cells activated might influence the disease progression of Gastric cancer (Figure 4C).

### WGCNA

**Figure 5:**
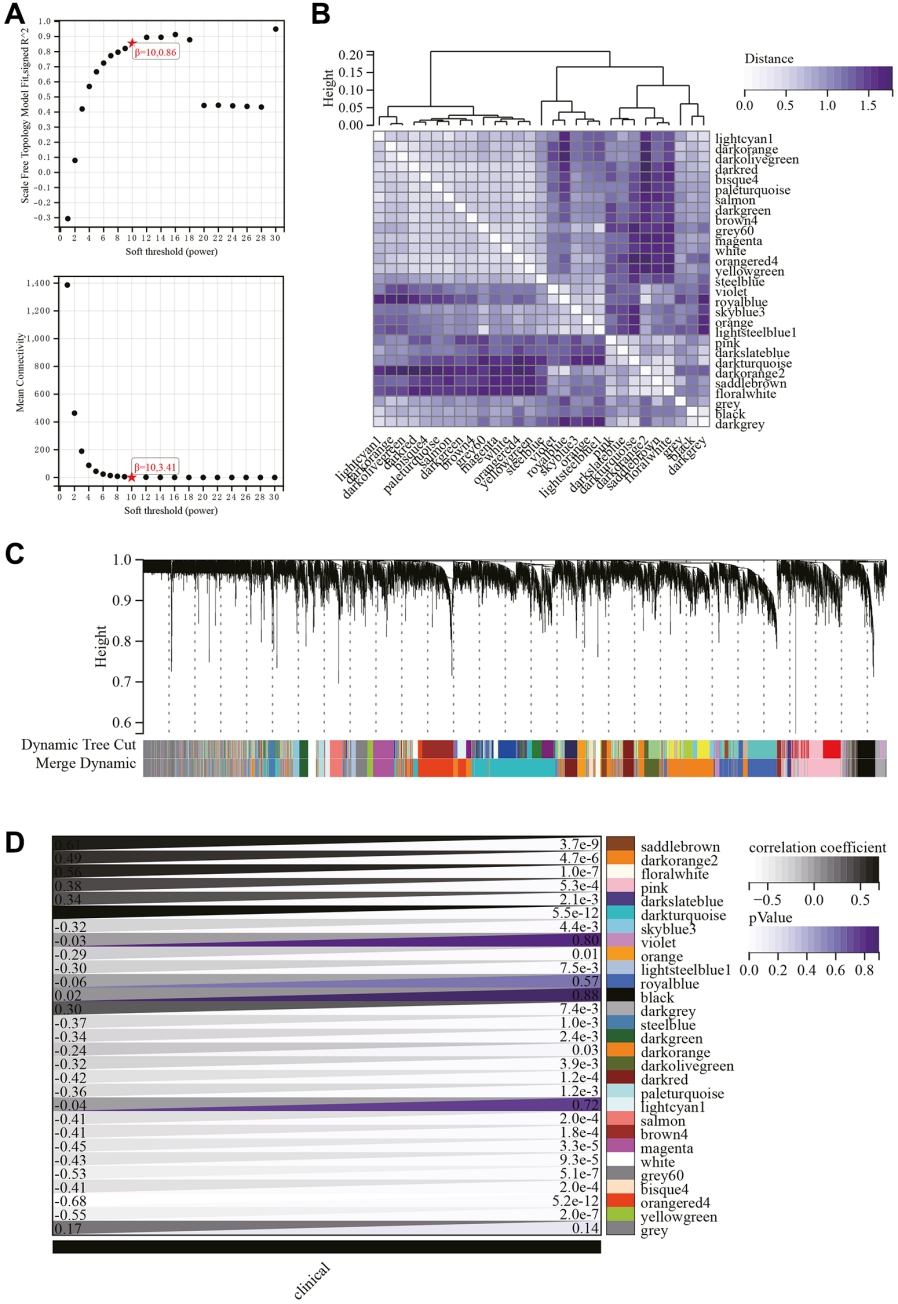
**WGCNA.** (**A**) β = 10, 0.86, β = 10, 3.41. (**B**) Gene hierarchical clustering tree. (**C**) Generated 29 modules. (**D**) Heatmap showing the correlation between modules and phenotypes.

**Figure 6:**
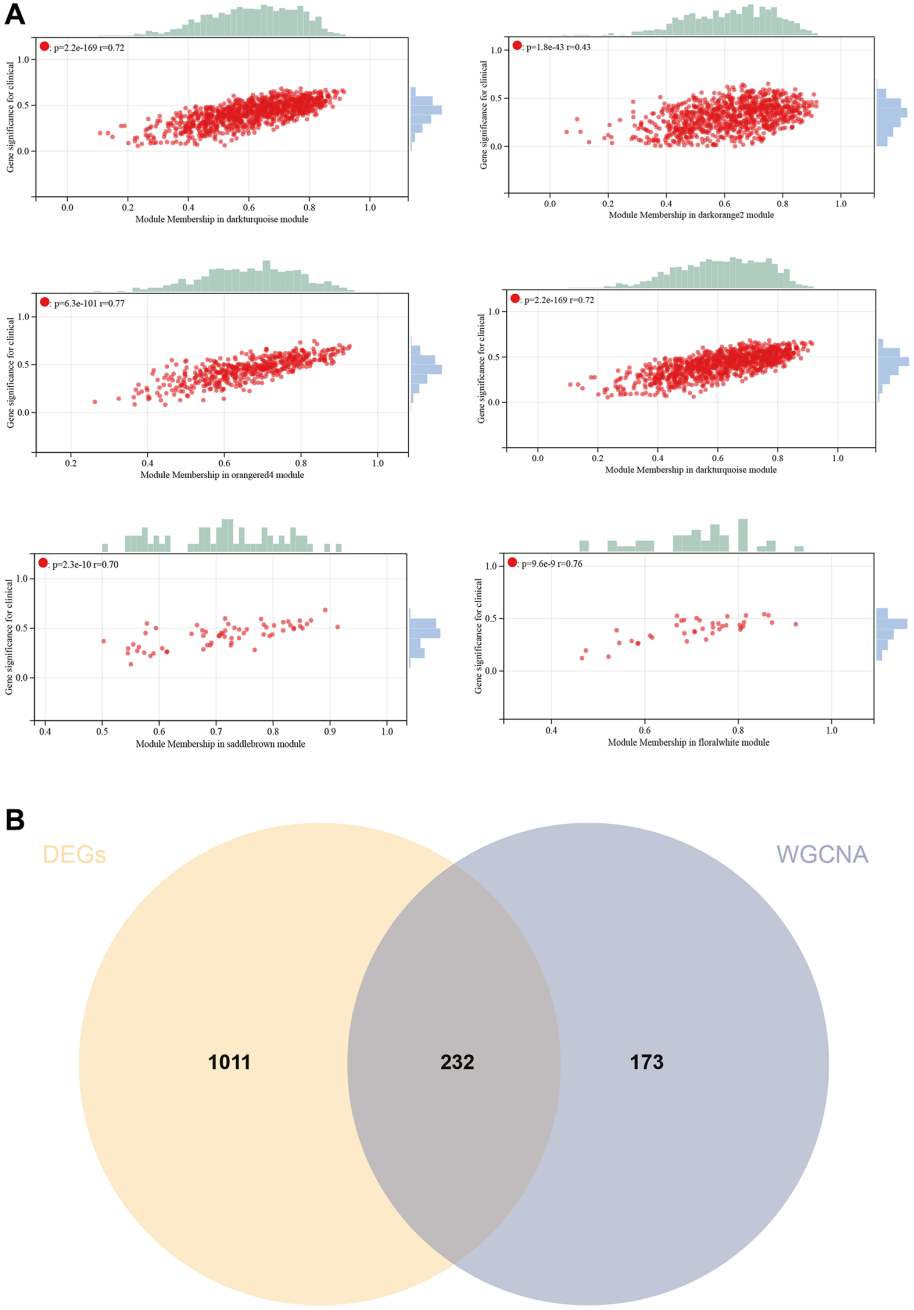
**WGCNA.** (**A**) GS to MM correlation scatter plot of associated hub genes. (**B**) Venn diagram.

The selection of the soft-thresholding power is an important step in WGCNA analysis. The network topology analysis determined a soft thresholding power of 10 (Figure 5A). A hierarchical clustering tree of all genes was constructed, and interactions between important modules were analyzed (Figure 5B). A total of 29 modules were identified (Figure 5C). Additionally, a module-phenotype correlation heatmap was generated (Figure 5D), as well as a scatter plot showing the correlation between GS and MM for relevant hub genes (Figure 6A).

Hub genes were identified by calculating the correlation of module eigengenes (MM) with gene expression. Using a cutoff criterion of |MM| >0.8, six highly connected genes were designated as hub genes in clinically significant modules. A Venn diagram of hub genes and DEGs was created for subsequent analysis (Figure 6B).

### Protein-Protein interaction (PPI) network construction and analysis

**Figure 7:**
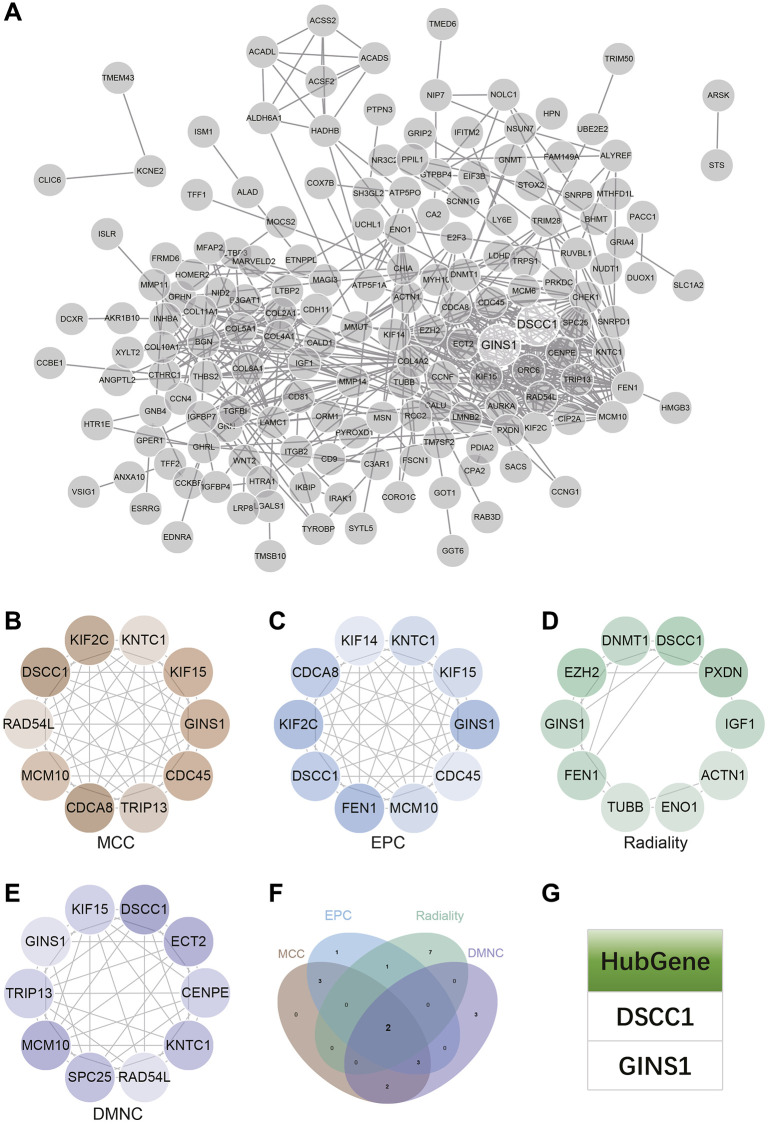
**Construction and analysis of protein-protein interaction (PPI) networks.** (**A**) The PPI network. (**B**–**E**) Identification of central genes using four algorithms (MCC, DMNC, EPC, Radiality). (**F**) Venn diagram to obtain the union as core genes. (**G**) Identification of 2 core genes (DSCC1, GINS1).

The PPI network for DEGs was constructed using the STRING online database and analyzed using Cytoscape software (Figure 7A). Four algorithms (MCC, DMNC, EPC, Radiality) were employed to identify central genes (Figure 7B–7E). A Venn diagram was used to obtain the intersection as core genes (Figure 7F), ultimately resulting in two core genes, DSCC1 and GINS1 (Figure 7G).

### Survival analysis

**Figure 8:**
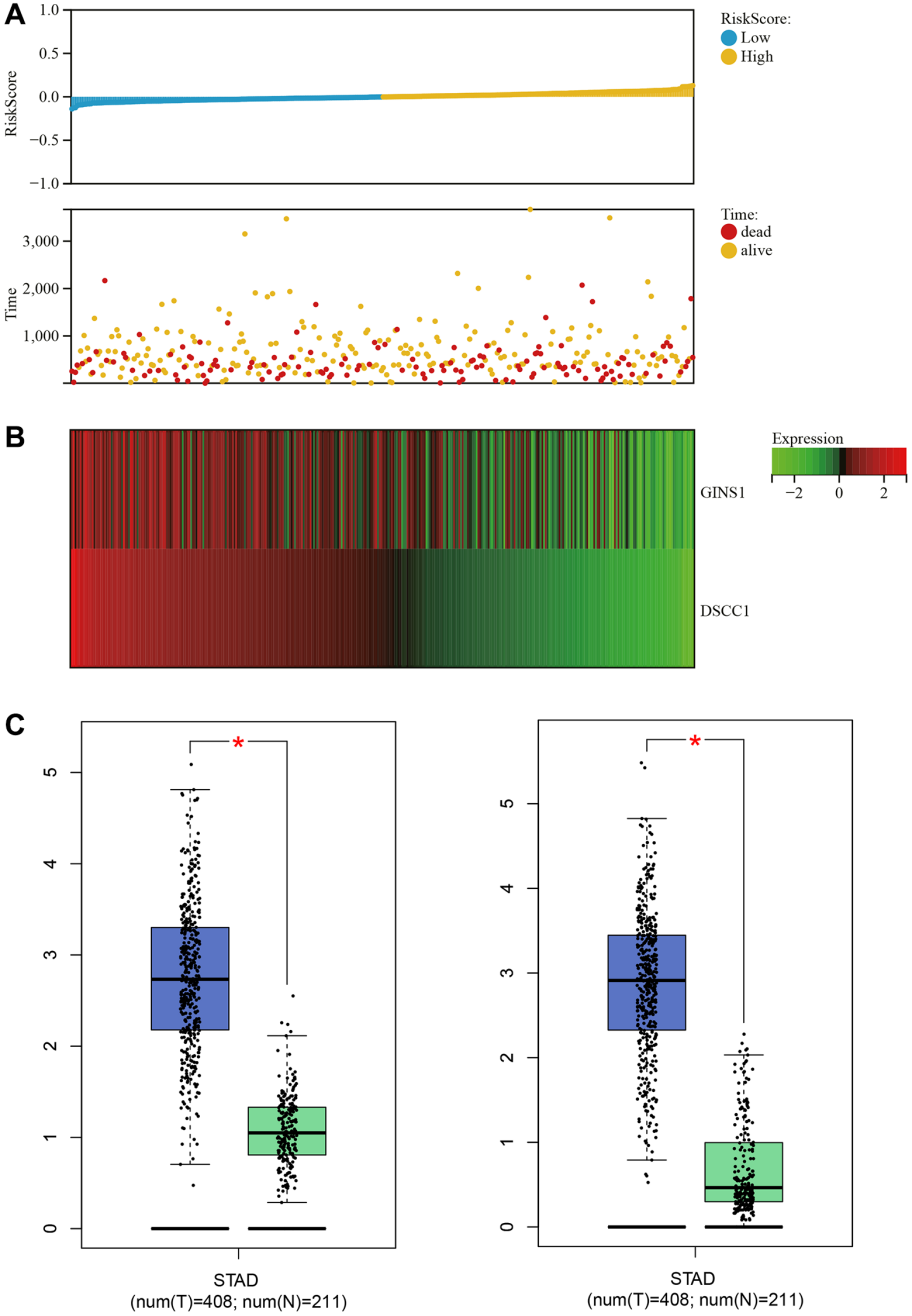
**Survival analysis.** (**A**) Relationship graph between prognosis scores. (**B**) Visualization of core gene expression in the gastric cancer survival data heatmap. (**C**) Boxplot results for core gene expression in gastric cancer.

Clinical survival data for Gastric cancer were downloaded from TCGA. The survival analysis revealed that as the risk score increased, the patient's survival rate significantly decreased. The low-risk group exhibited significantly higher survival time and rate compared to the high-risk group (Figure 8A). A heatmap of core gene expression in Gastric cancer survival data indicated that the core genes, DSCC1 and GINS1, acted as protective factors. As the risk score increased, their expression showed a downregulation trend (Figure 8B).

Box plots of core gene expression in Gastric cancer and normal samples demonstrated differential expression of core genes (DSCC1 and GINS1), with higher expression in Gastric cancer samples and lower expression in normal samples (Figure 8C).

### Core gene expression heatmaps

**Figure 9:**
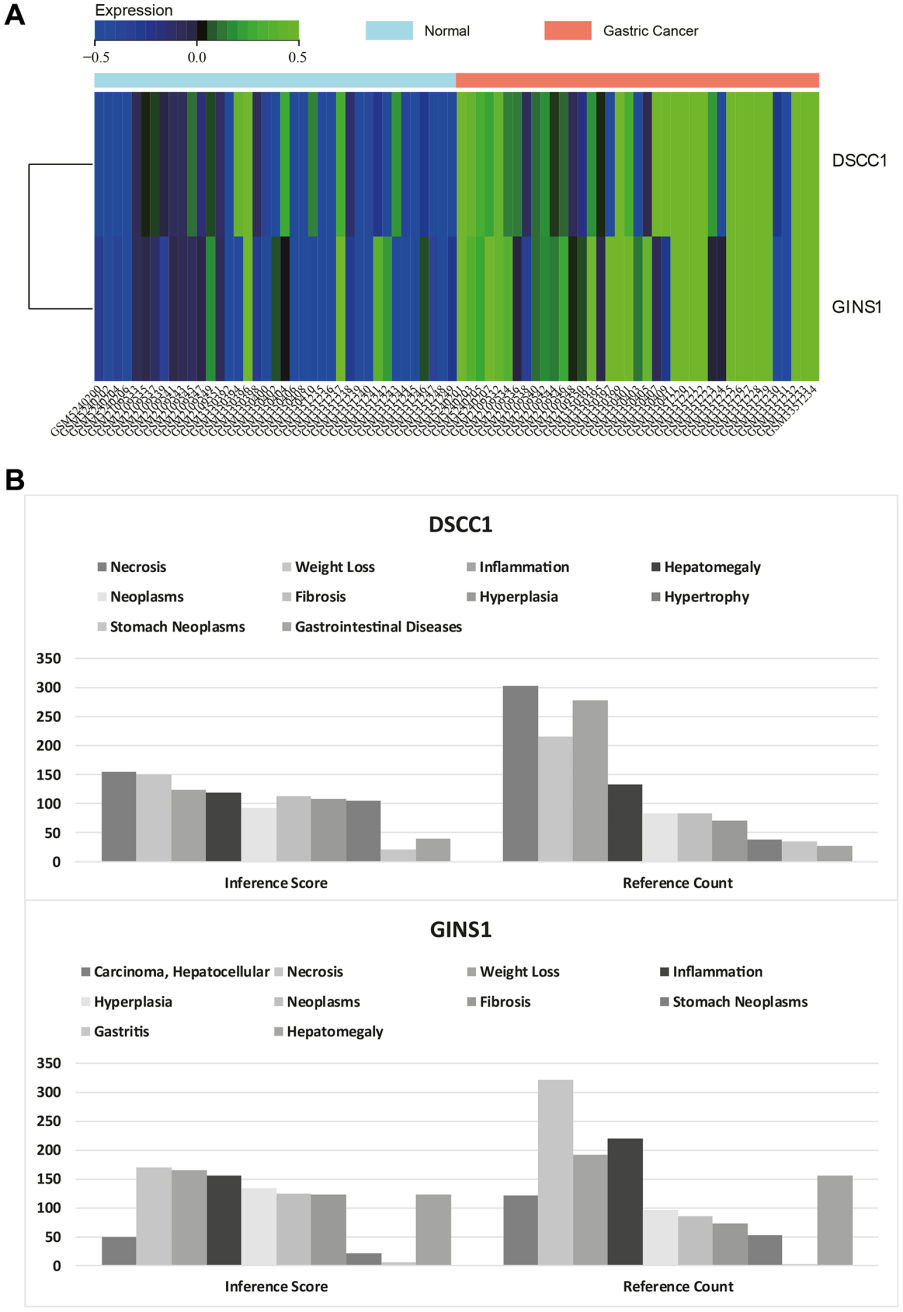
**Heatmap of core gene expression.** (**A**) Visualization of core gene expression in the merged matrix of gastric cancer datasets GSE79973, GSE118916, GSE118897, GSE172032, with respective heatmaps. (**B**) CTD analysis showing associations of core genes (DSCC1, GINS1) with gastric tumors, gastrointestinal diseases, tumors, gastritis, inflammation, and necrosis.

Heatmaps visualizing the expression of core genes in the merged matrices of Gastric cancer datasets GSE79973, GSE118916, GSE118897, and GSE172032 were generated (Figure 9A). These heatmaps indicated higher expression of the core genes (DSCC1 and GINS1) in Gastric cancer samples compared to normal samples. Based on these results, it is hypothesized that these core genes may play a regulatory role in Gastric cancer.

### CTD analysis

In this study, the list of hub genes was input into the CTD website to identify diseases associated with core genes, improving our understanding of the gene-disease associations. The core genes (DSCC1 and GINS1) were found to be associated with gastric neoplasms, gastrointestinal diseases, tumors, gastritis, inflammation, and necrosis (Figure 9B).

### Prediction and functional annotation of miRNAs related to hub genes

In this study, the list of hub genes was input into TargetScan to identify miRNAs that might regulate these genes, enhancing our understanding of gene expression regulation. We found that the miRNA related to GINS1 is hsa-miR-205-5p, while the miRNA related to DSCC1 is hsa-miR-325-3p (Table 1).

**Table 1:** A summary of miRNAs that regulate hub genes.

	**Gene**	**MIRNA**
**1**	**GINS1**	hsa-miR-205-5p
**2**	**DSCC1**	hsa-miR-325-3p

### DSCC1 and GINS1 expression levels

#### DSCC1 and GINS1 protein expression levels

**Figure 10:**
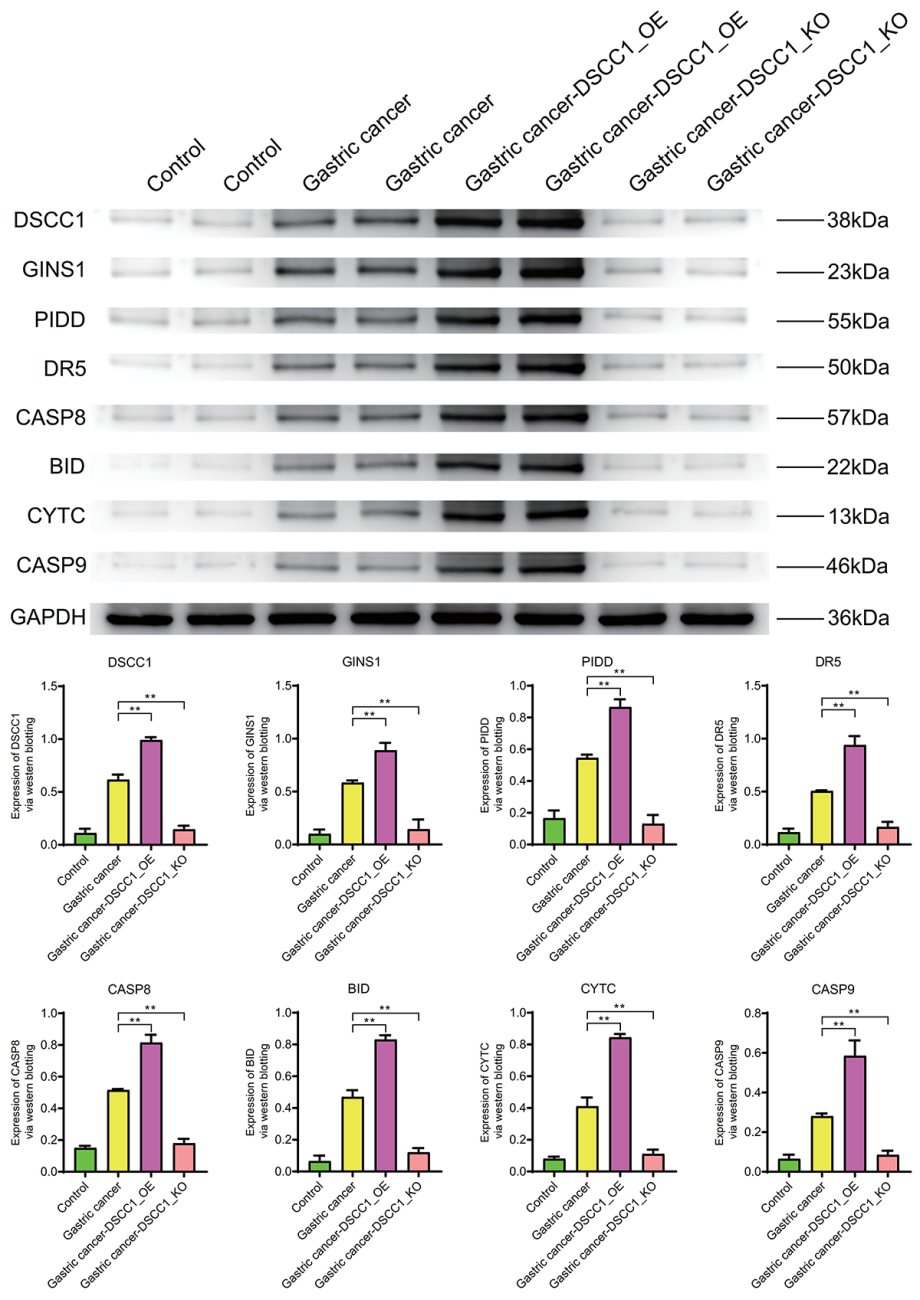
**Western blotting.** Protein expression levels of DSCC1, GINS1, PIDD, DR5, CASP8, BID, CYTC, CASP9.

WB results show that the expression levels of DSCC1, GINS1, PIDD, DR5, CASP8, BID, CYTC, CASP9 in the Gastric cancer group are significantly higher than in the Control group (*P* < 0.01). In the Gastric cancer-OE group, their expression levels are significantly higher than in the Gastric cancer group. In the Gastric cancer-KO group (*P* < 0.01), their expression levels are significantly lower than in the Gastric cancer group (*P* < 0.01) (Figure 10).

**Figure 11:**
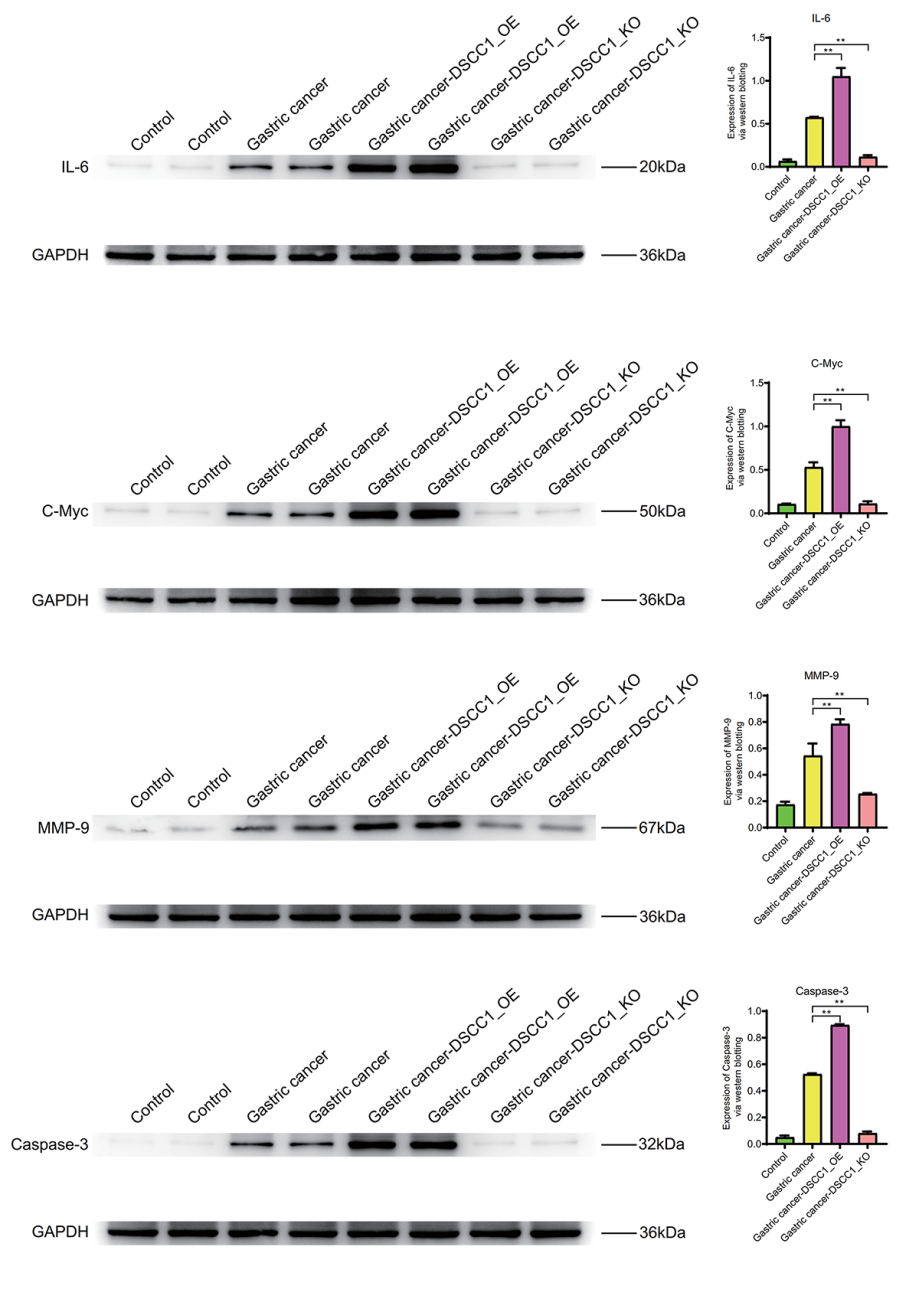
**Western blotting.** Protein expression levels of IL-6, C-Myc, MMP-9, Caspase-3.

Inflammatory molecule IL-6, cell cycle regulator C-Myc, cell migration molecule MMP-9, and apoptosis molecule Caspase-3 exhibit significantly higher expression levels in the Gastric cancer group compared to the Control group (*P* < 0.01). In the Gastric cancer-OE group (*P* < 0.01), their expression levels are significantly higher than in the Gastric cancer group. In the Gastric cancer-KO group, their expression levels are significantly lower than in the Gastric cancer group (*P* < 0.01) (Figure 11).

#### The mRNA expression levels of DSCC1 and GINS1

**Figure 12:**
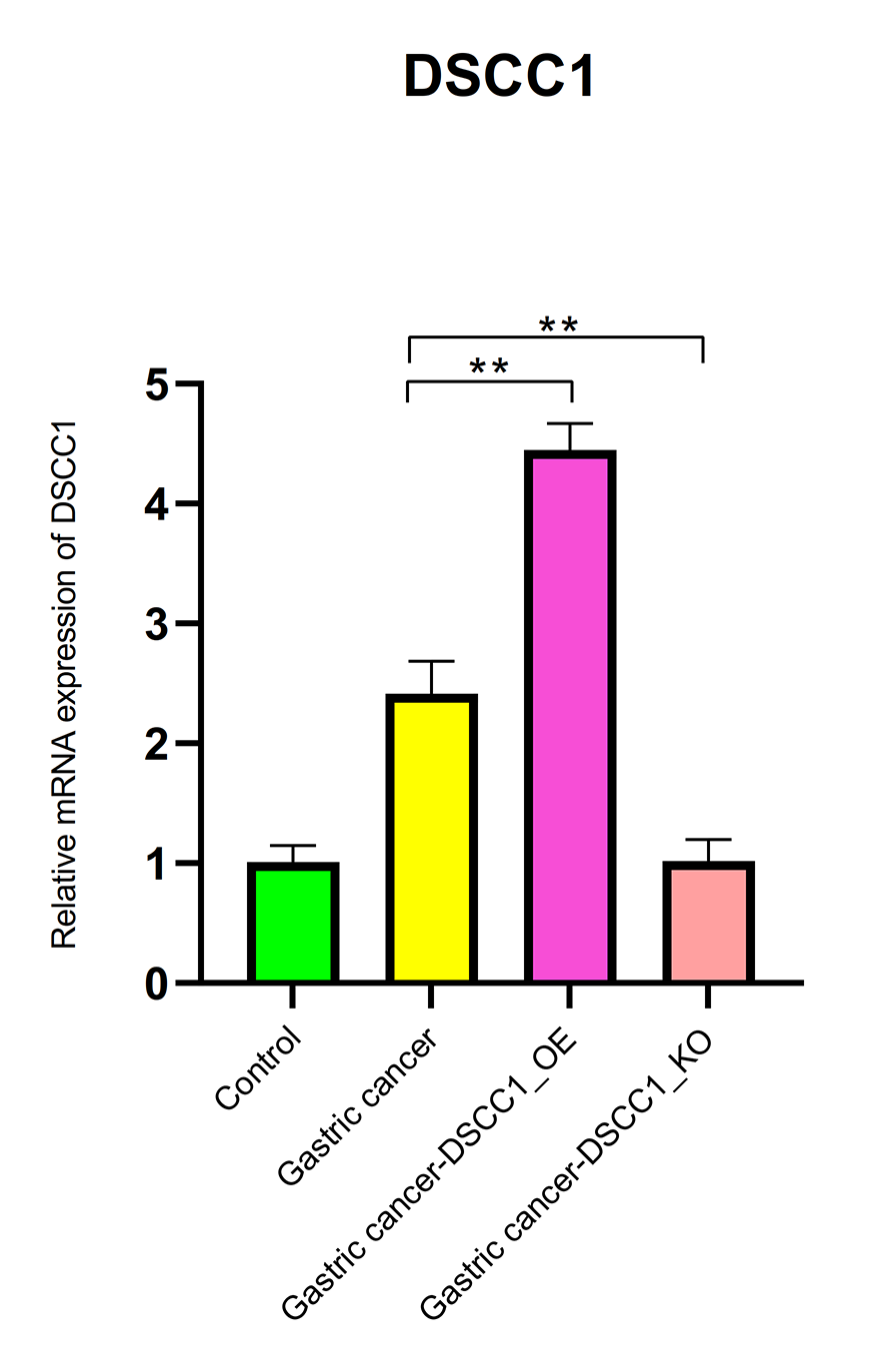
**Reverse transcription-polymerase chain reaction.** mRNA expression levels of DSCC1.

**Figure 13:**
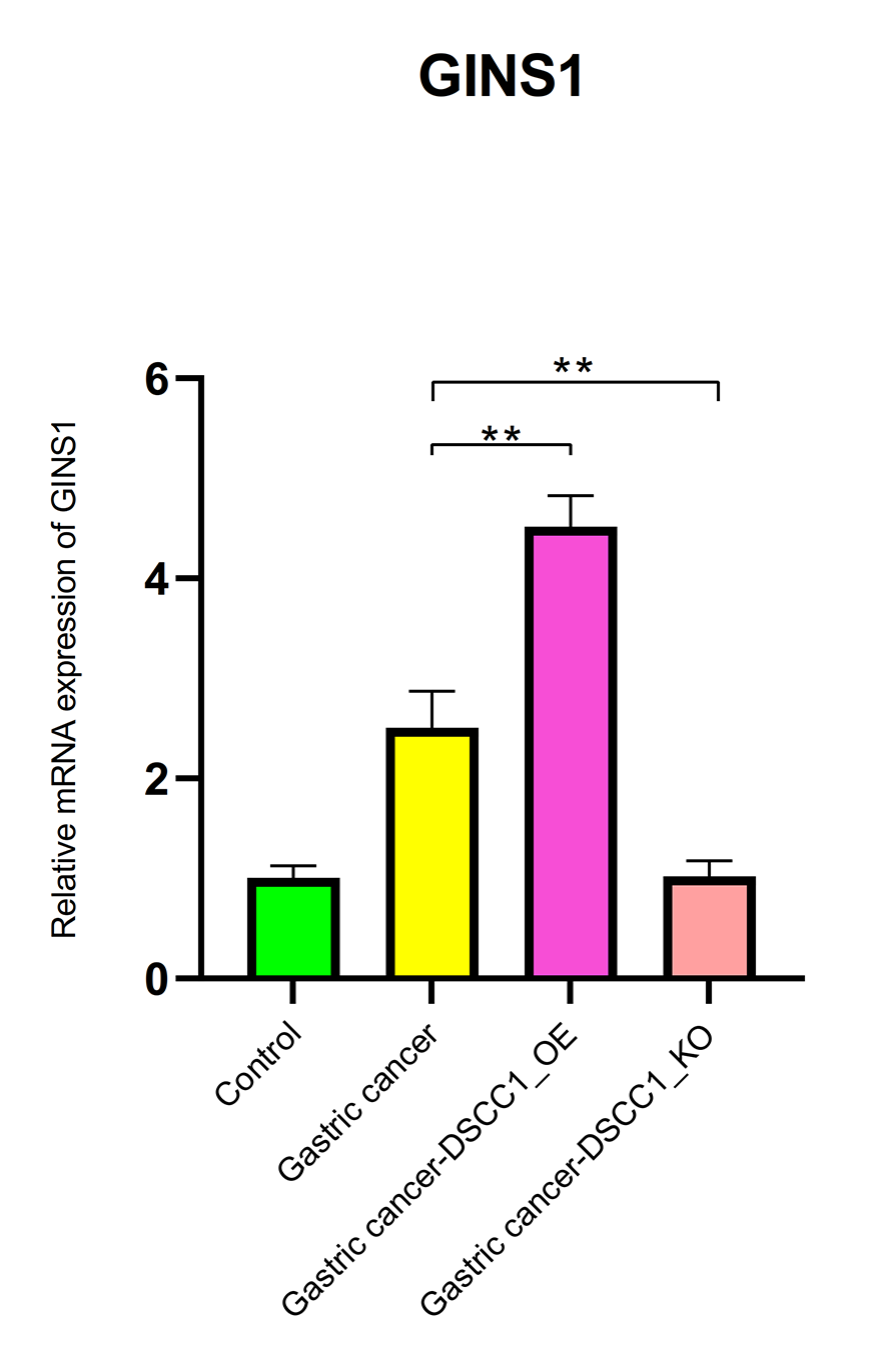
**Reverse transcription-polymerase chain reaction.** mRNA expression levels of GINS1.

The RT-PCR results showed that, compared to the Control group, the mRNA expression levels of DSCC1 and GINS1 in the Gastric cancer group were significantly increased (*P* < 0.01). In the Gastric cancer-OE group, the mRNA expression levels of DSCC1 and GINS1 were significantly higher than in the Gastric cancer group (*P* < 0.01). In the Gastric cancer-KO group, the mRNA expression levels of DSCC1 and GINS1 were significantly lower than in the “Gastric cancer” group (*P* < 0.01) (Figures 12, 13).

## DISCUSSION

Gastric cancer is a highly lethal disease that, without early diagnosis and treatment, can quickly spread to adjacent tissues and other organs, posing a grave threat to patients’ lives [11]. Researchers have focused on the role of CT in accurately staging gastric cancer, determining the extent of tumor invasion, lymph node involvement, and the presence of distant metastases. Late-stage Gastric cancer presents significant treatment challenges and often results in low cure rates. The symptoms of Gastric cancer significantly impact patients’ quality of life, severely affecting their dietary habits and physical health, including issues such as indigestion, upper abdominal discomfort, loss of appetite, weight loss, vomiting, and black stools [12, 13]. The treatment of Gastric cancer can be financially burdensome, involving costly procedures such as surgery, chemotherapy, radiation therapy, and drug treatments, potentially imposing substantial economic burdens on patients and their families [14]. Furthermore, Gastric cancer patients often experience psychological burdens, including anxiety, depression, and social isolation. Given the severity of Gastric cancer’s impact, an in-depth exploration of its molecular mechanisms is crucial for a better understanding of disease progression and the development of more effective treatment methods [15]. Although CT plays a crucial role in the diagnosis and management of gastric cancer, there are still limitations and shortcomings, including: Limitations in Early Lesion Detection: CT has relatively low sensitivity for early-stage gastric cancer. Small lesions, early infiltrative growth stages, and submucosal infiltration may not be easily detected through routine CT scans. Limited Histological Information: CT provides anatomical information, but details regarding differentiation, histological types, and other aspects are limited. For a more comprehensive assessment of tumor characteristics, additional tests such as endoscopy or tissue biopsy may be needed.

The findings of this study reveal that DSCC1 and GINS1 are overexpressed in Gastric cancer, and higher expression levels are associated with worse patient outcomes, suggesting that these two genes may play pivotal roles in Gastric cancer. These target genes likely contribute to the regulation of DNA replication, cell division, and chromosome stability [16, 17]. This research offers a foundation for the development of targeted therapies and personalized treatment strategies, aiming to improve the treatment and prognosis of Gastric cancer patients. Additionally, it paves the way for exciting avenues in future cancer research, fostering a better understanding of tumor molecular mechanisms and the development of novel therapeutic approaches.

DNA Replication and Sister Chromatid Cohesion 1 (DSCC1) is a gene or protein associated with DNA replication and the cohesion of sister chromatids, playing a significant role in cell biology [8, 18]. DSCC1 collaborates with other proteins, forming a complex connector known as the Cohesin complex, which assists in keeping sister chromatids together during cell division—a crucial step in ensuring the correct separation of chromosomes into newly formed cells [19]. Disruption of Cohesin complex function may result in abnormal chromosome separation, leading to chromosome instability and other cellular biology issues [20]. Research suggests that abnormal expression of DSCC1 may be linked to the development of certain cancers, as seen in a study related to lung adenocarcinoma [21]. Notably, adenocarcinoma is the most common type of Gastric cancer, typically originating in the glandular cells of the stomach mucosa.

DSCC1’s expression is dynamic throughout various stages of cell division, playing a role during the S phase (DNA replication phase) and M phase (mitosis), assisting in chromosome replication and sister chromatid cohesion [22]. Aberrant DSCC1 function is associated with diseases, particularly those characterized by chromosome instability and rearrangements. For example, a study found that DSCC1, along with POLE3, interacts with the catalytic subunit of polymerase ε, potentially affecting DNA replication and emphasizing the importance of these factors in parallel pathways for leading strand DNA replication [23]. Similarly, in colorectal cancer, it was observed that DSCC1 is overexpressed in tumor tissues, and higher cytoplasmic DSCC1 expression in tumor areas is associated with lower survival rates (*P* = 0.047), further validated in a study by Kim et al. [24]. DSCC1 is identified as a crucial component of the CTF18-RFC module, highly correlated with the growth and metastasis of colon cancer cells.

GINS complex subunit 1 (GINS1) is a component of the GINS complex, a protein complex consisting of GINS1, GINS2, GINS3, and GINS4. The GINS complex plays a vital role in the initiation and maintenance of DNA replication [25, 26]. Working in coordination with DNA polymerase, the GINS complex ensures the smooth progression of DNA strand replication, and GINS1, along with its related subunits, performs specific functions within the DNA replication complex, assisting in DNA unwinding, replication, and synthesis. This is critical in ensuring that newly formed cells have accurate genetic information after each cell division [27]. GINS1’s expression is related to different stages of the cell cycle, especially during the S phase (DNA replication) and M phase (mitosis), where it plays an essential role in aiding DNA replication and ensuring proper chromosome separation [28]. Dysfunctional GINS1 may lead to DNA replication errors, chromosome instability, and cell cycle issues, highly relevant to various diseases, including cancer.

Studies have found that GINS1 is downregulated in one subtype of human cancer and typically upregulated in 23 different subtypes, with the upregulation of GINS1 significantly correlating with poorer overall survival in liver hepatocellular carcinoma (LIHC), lung adenocarcinoma (LUAD), and kidney renal clear cell carcinoma (KIRC) [29]. Results from Yang et al. [30] further validate the upregulation of GINS1 expression in glioblastoma cells and tissues. The analysis reveals that GINS1 promotes glioblastoma cell proliferation and migration through the mediation of USP15 and TOP2A deubiquitination.

The literature review presented aligns with our findings that DSCC1 and GINS1 are overexpressed in Gastric cancer, and higher expression levels are associated with poor prognosis. These two proteins are key players in DNA replication and cell cycle regulation, working in synergy with other replication proteins to ensure accurate DNA replication and cell division. Consequently, DSCC1 and GINS1 are likely to play important roles in the development of Gastric cancer. Studying the role of DSCC1 and GINS1 in clinical gastric cancer samples and their relationship with patient prognosis is of great significance for understanding the pathogenesis of gastric cancer and developing potential therapeutic strategies, which will help to more accurately evaluate the value of these two genes as potential therapeutic targets. This research has far-reaching societal and scientific implications, impacting human life, health, and healthcare. It holds broad potential clinical applications to significantly improve the quality of patients’ lives, extend lifespans, and provide more effective treatment options, driving continuous advancements in healthcare. It aids in the discovery of new biomarkers and diagnostic methods for early disease detection. It contributes to achieving precision personalized medicine by tailoring treatment plans for each individual based on their genotype, lifestyle, and medical history, incorporating applications in genomics, transcriptomics, and proteomics to understand individual biological characteristics. Medical research findings also closely relate to targeted therapy. By unraveling the molecular mechanisms of diseases, it facilitates the design and development of drugs targeting specific molecular targets. It helps understand the mechanisms of drug resistance and develop strategies to overcome it. Furthermore, it offers biomarkers for monitoring treatment effectiveness, evaluating patient responses, adjusting treatment plans, and intervening early when necessary.

While this study conducted rigorous bioinformatic analyses, further validation of their functions through *in vivo* experiments, such as gene overexpression or knockout studies in animals, is warranted. In future research, in-depth exploration in this direction should be considered.

In summary, DSCC1 and GINS1 are overexpressed in gastric cancer, and higher expression levels are associated with worse prognosis. DSCC1 and GINS1 might be the complementary biomarkers of computed tomography for diagnostic grading of gastric cancer. These genes primarily function in cell cycle regulation, macrophage migration, protein complex binding, P53 signaling pathways, protein digestion and absorption, and metabolic pathways, suggesting their regulatory roles in Gastric cancer. In the future, in-depth exploration of the functions of organisms will be conducted to better understand the biological and physiological basis and provide more comprehensive knowledge for the treatment of diseases and health maintenance. Research directions focus on elucidating the functions of molecules, cells, organs, and physiological systems, thereby driving advances in medicine and biology. The process of translating research findings into clinical practice to assess their efficacy, safety, and practical applicability. Translating new treatments, drugs, diagnostic tools, and treatment strategies into medical practice. Conduct clinical trials to evaluate the efficacy of new treatments or drugs. Clinical observational studies can also be conducted to assess the effectiveness of new methods by looking at treatment outcomes in large patient populations.
